# Study on Preparation and Separation and Adsorption Performance of Knitted Tube Composite β-Cyclodextrin/Chitosan Porous Membrane

**DOI:** 10.3390/polym11111737

**Published:** 2019-10-24

**Authors:** Qian Tang, Nana Li, Qingchen Lu, Xue Wang, Yaotian Zhu

**Affiliations:** 1 School of Textile Science and Engineering, Tiangong University, No. 399 Binshui Xi Road, Xiqing District, Tianjin 300387, China; 1730011043@stu.tjpu.edu.cn (Q.T.); luqingchen@stu.tjpu.edu.cn (Q.L.); 1730011073@stu.tjpu.edu.cn (X.W.); 1731015034@stu.tjpu.edu.cn (Y.Z.); 2 State Key Laboratory of Separation Membranes and Membrane Processes, Tianjin Polytechnic University, Tianjin 300387, China

**Keywords:** porous membranes, adsorption performance, chitosan, β-cyclodextrin, crosslinking

## Abstract

In order to obtain membranes with both organic separation and adsorption functions, knitted tube composite β-cyclodextrin/chitosan (β-CD/CS) porous membranes were prepared by the non-solvent induced phase separation (NIPS) method using CS and β-CD as a membrane-forming matrix, glutaraldehyde as crosslinking agent to improve water stability, and knitted tube as reinforcement to enhance the mechanical properties. Fourier transform infrared spectroscopy (FTIR), scanning electron microscopy (SEM), contact angle, water flux, bovine serum albumin (BSA) rejection and tensile test were carried out. The FTIR demonstrated that the β-CD and CS had been successfully crosslinked. With the crosslinking time increased, the membrane structure became denser, the contact angle and the rejection rate increased, while the water flux decreased. The strength and elongation at a break were 236 and 1.7 times higher than these of bare β-CD/CS porous membranes, respectively. The strength of crosslinking membranes increased further. The adsorption performance of composite membranes was investigated for the removal of phenolphthalein (PP) from aqueous solution. The adsorption process followed the Langmuir isotherm model, and the kinetic behavior was accorded with the Double constant equation and the Elovich equation. The adsorption mechanism could be explained by the synergistic effect of host-guest interaction from β-cyclodextrin, non-uniform diffusion and porous network capture.

## 1. Introduction

The problem of water pollution has become increasingly prominent due to the rapid development of the modern industry. Over time, a large number of sewage treatment technologies have emerged. Among them, the membrane separation technology has caused widespread concern because of its simple operation and high efficiency [1,2].

**Figure 1:**
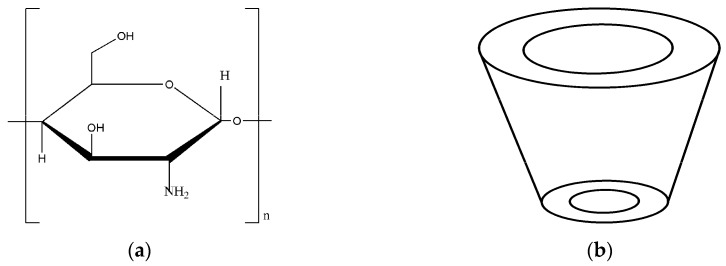
Structure of (**a**) Chitosan (CS) molecule and (**b**) β-CD molecule.

Chitosan (CS) is the N-deacetylated product of chitin, and the –OH and –NH_2_ containing in the molecule can form a chelate with metal ions (Figure 1a) [3], which is widely used in metal ions adsorption [4,5,6], organic adsorption [7,8] and other water treatment fields. In addition, CS possess good membrane formation, excellent biocompatibility and hydrophilicity making it widely used in the membrane separation process [9,10,11]. Rodrigo et al. [12] prepared CS membranes by glutaraldehyde crosslinked to adsorb Hg^2+^, and the maximum amount of adsorption was 75.7 mg/g at pH 6. The CS porous membranes made by Qin et al. [13] which rejection of methyl blue more than 99%. When the additive was 0.5 g/L, the adsorption capacity of Cr^6+^ was 168.41 mg/g, after crosslinking increased to 181.60 mg/g.

β-cyclodextrin (β-CD) molecule is composed of 7 glucose monomers connected by α-1,4-glycoside bond. β-CD owns a cone-shaped three-dimensional ring structure, which consist of a hydrophilic exterior and a hydrophobic interior cavity that can form inclusion complexes with guest (organic) molecules, depending on the size and polarity of the β-CD (Figure 1b) [14]. β-CD have been extensively adopted as the adsorbent towards organic compounds due to its lower cost and moderate cavity size [15,16]. Tan et al. [17] prepared graphene/β-CD (GNS/β-CD) adsorbent. Its saturated adsorption capacities of methyl blue, methyl orange and basic fuchsine were 580.4, 328.2 and 425.8 mg/g, respectively, which was a fast and efficient adsorbent. Ying et al. [18] modified the β-CD polymer with Fe_3_O_4_ magnetic nanoparticles to adsorb deoxyribonucleic acid (DNA). The results showed that the interaction between the adsorbent and DNA was achieved by inclusion formation, hydrophobic interaction, hydrogen bonding, and van der Waals forces. Junthip et al. [19] crosslinked β-CD with citric acid to adsorb paraquat from water, and found that its adsorption isotherm conformed to the Langmuir model, and the adsorption kinetics accorded with the Pseudo-second order model. After four cycles of regeneration, the regeneration rate was 78.3%. Jiang et al. [20] synthesized a novel crosslinked porous β-CD-based polymer containing carboxylic acid groups, which has a triple absorption effect of inclusion, porous network capture and electrostatic interaction. The maximum adsorption capacity of methylene blue dye was 672 mg/g.

Based on the dual characteristics of the adsorption performance of CS and the inclusion of β-CD, the researchers combine the prominent properties of both materials has attracted much attention. Preethi et al. [21] combined CS/β-CD with lanthanum to adsorb CrO_4_^2−^ and F^−^ which the maximum adsorption capacity of 97.67 and 8.44 mg/g, respectively, and the maximum recycling times of five times. Zhao et al. [22] prepared a tri-functional adsorbent of CS-ethylene diamine tetraacetic acid (EDTA)-β-CD, founding that the highest adsorption capacity of toxic metal Pb^2+^ and Cd^2+^ were 0.803 and 1.2558 mmol/g, respectively. When it came to bisphenol-S, ciprofloxacin, procaine and imipramine there also had good adsorption efficiency. Adwalai et al. [23] modified β-CD with amino and hydroxyl groups in CS to prepare a nanocomposite membranes composed of functionalized β-CD and polyethersulfone (PES) for treating low pressure wastewater. The removal efficiency of heavy metal ions Cr^6+^, Zn^2+^, Fe^2+^ and Cd^2+^ were 92%, 90%, 82% and 87%, respectively. Using maleic chain as a bridge and glutaraldehyde as crosslinking agent, Jiang et al. [24] synthesized CS-CD complex. It was found that the optimal adsorption capacity of methyl orange was 392 mg/g. Its adsorption mechanism could be explained by the electrostatic attraction of the amino group in CS and the synergistic interaction between β-CD and the guest.

Given that combination of CS and CD can greatly improve the adsorption performance. This study prepared knitted tube composite β-CD/CS porous membranes using glutaraldehyde as a cross-linker to further increase the structure stability. The porous membrane structure, separation performance, mechanical properties and adsorption performance were discussed. Meanwhile, the kinetics and isotherm towards the adsorption of phenolphthalein (PP) and the adsorption mechanism was investigated.

## 2. Materials and Methods

### 2.1. Materials

Knitted tube (L185 reverse support tube) is made of polyester yarn, purchased from Zhengze New Energy Materials Technology Co., Ltd. (Anhui, China). CS and BSA (biological reagent) were purchased from Sinopharm Chemical Reagent Beijing Co., Ltd. (Beijing, China). β-CD, NaOH and 50% glutaraldehyde were supplied by Damao Chemical Reagent Factory (Tianjin, China). Polyethylene glycol (PEG, molecular weight =600,) was ordered from Tianjin Ruijinte Chemicals Co., Ltd. (Tianjin, China). Phenolphthalein (PP), acetic acid, anhydrous sodium carbonate and glycerol were provided by Kemiou Chemical Reagent Co., Ltd. (Tianjin, China). Sodium bicarbonate was offered by Yingdaxigui Chemical Reagent Factory (Tianjin, China). All the chemicals and reagents were of analytical grade without further purification. Deionized water was made in the laboratory.

### 2.2. Preparation of Knitted Tube Composite β-CD/CS Porous Membranes

**Figure 2:**
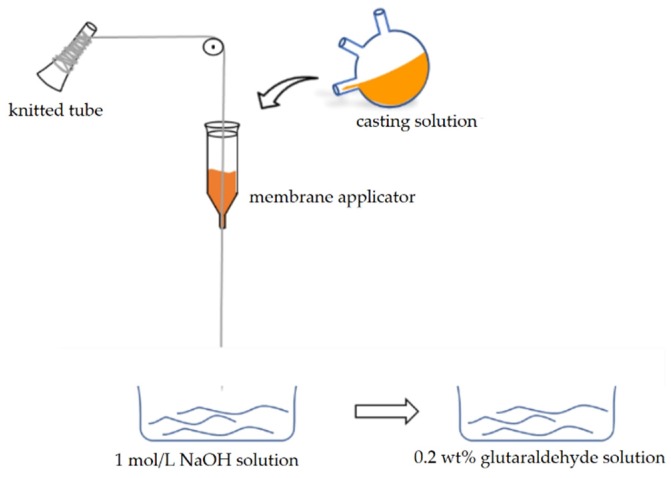
Schematic diagram of membranes preparation process.

First, a certain mass of CS was weighed and dissolved in 2 vol % acetic acid solution to acquire 7 wt % CS solution, then 3 g PEG and 3.5 g β-CD were successively added and stirred at room temperature for 2 h. The resulting solution was defoamed in the stirring defoaming apparatus (MAZERUSTAR KK–250S, Kurabo Industries Ltd., Osaka, Japan) to obtain uniform casting solution. Then, the casting solution was evenly coated on the knitted tube using a laboratory-made membrane applicator. After the obtained membranes were soaked in 1 mol/L NaOH solution for precipitation and immersed in 0.2 wt % glutaraldehyde solution for crosslinking. The crosslinking time was 10 s, 30 s, 60 s, 90 s, 3 min, 6 min, 10 min, 20 min, 30 min, 60 min and 90 min, respectively. Finally, the membranes were repeatedly washed with distilled water to reach the neutral pH environment, and the knitted tube composite β-CD/CS porous membranes were prepared. The membranes were soaked with glycerol and dried before the next tests, except for the natural drying of the membrane during the contact angle test. The preparation procedure of the membranes was illustrated in Figure 2.

### 2.3. Characterization

#### 2.3.1. FTIR Analysis

The infrared spectrum of CS powder, CD powder and the β-CD/CS porous membrane were obtained by a Fourier Transform Infrared Spectrometer (Nicolet iS50, Thermo Fisher Scientific Co., Ltd., Waltham, MA, USA). The wave number range was 400–4000 cm^−1^.

#### 2.3.2. SEM Analysis

The pretreatment procedures on the pore size is important, since it determines the performance characteristics [25]. The β-CD/CS porous membranes were soaked with glycerol and dried to maintain the structure of the membrane pores and prevent the pores from collapsing [26]. Epoxy was firstly wrapped on the sample preparation to prevent the membrane layer from falling off the knitted tube during brittle breaking process, and then cut off directly after liquid nitrogen treatment. The morphology was observed by desktop scanning electron microscope (Phenom pure, Funer Scientific Instrument Co., Ltd, Eindhoven, Netherlands) after gold sputtering.

#### 2.3.3. Mechanical Properties Test

The tensile properties of the knitted tube composite β-CD/CS membranes and bare β-CD/CS membranes were examined by a universal mechanical tester (3369, Instron, Boston, MA, USA) with a clamp spacing of 50 mm and a tensile rate of 50 mm/min. The tensile strength was calculated for five times independently, and the average value was obtained.

#### 2.3.4. Contact Angle Test

The static water contact angles of the membrane surfaces were measured by a video optical contact angle measuring instrument (OCA15pro, Dataphysics company, Filderstadt, German) at room temperature (25 ± 1) °C in air. The average value of each membrane was calculated with five different locations [27].

#### 2.3.5. Water Flux Test

The pure water flux of the membranes was measured by a laboratory-made device. The membranes were immersed in pure water for 48 h before the test. The membrane was pre-pressed at a pressure of 0.15 MPa for 20 min, and then tested at a pressure of 0.1 MPa for 30 min. The results were calculated by Equation (1):

(1) $$J=\frac{V}{A\times t}$$

where *J* (L/(m^2^·h)) is water flux. *V* (L) is the volume of permeated water. *A* (m^2^) is the effective area of the membranes and *t* (h) is the operation time.

#### 2.3.6. Rejection Rate Test

The BSA rejection test of the β-CD/CS porous membranes was characterized using 1 g/L BSA solution. The experiment was carried out at 0.1 MPa with the temperature at (25 ± 1) °C and the permeate was collected for 10 min. The BSA concentration in feed and permeate was determined by an ultraviolet-visible spectrophotometer (Evolution 201, Thermo Fisher Scientific Co., Ltd., Waltham, MA, USA) at 280 nm. The rejection rate was obtained by Equation (2):

(2) $$R=\frac{C_{o}-C_{e}}{C_{o}}\times 100\%$$

where *R* (%) is the rejection rate. *C_o_* (mg/L) and *C_e_* (mg/L) are the concentration of feed and permeation solutions, respectively.

### 2.4. Adsorption Experiments

There were 2, 4, 6, 8 and 10 mg/L PP solutions accurately prepared. The solution was measured with the ultraviolet-visible spectrophotometer with an incident wavelength of 553 nm, and the standard curve was drawn. The adsorption experiments of PP on membranes were carried out using batch methods. In a typical experiment, 0.1 g β-CD/CS porous membranes were immersed in 20 mL PP solution prepared above and standing for a period of time, then the β-CD/CS porous membranes were taken out to determine the absorbance of the filtrate. The effect of crosslinking time (10 s–90 min), adsorption time (5–120 min) and initial concentration of PP solution (0.5–4.5 mg/L) to the adsorption of β-CD/CS porous membranes were investigated, respectively. Adsorption capacity and adsorption efficiency were determined based on the Equations (3) and (4):

(3) $$q_{e}=\frac{(C_{o}-C_{e})\times V}{m}$$

(4) $$E=\frac{C_{o}-C_{e}}{C_{o}}\times 100\%$$

where *q_e_* (mg/g) is the adsorption capacity of the membranes at equilibrium. *E* (%) is adsorption efficiency. *C_o_* (mg/L) and *C_e_* (mg/L) are the initial and equilibrium concentration of PP solution, respectively. *V* (mL) is the volume of PP solution and *m* (g) is the mass of the porous membranes.

### 2.5. Adsorption Isotherms

PP solutions with concentrations of 1.0, 1.5, 2.0, 2.5, 3.0, 3.5, 4.0 and 4.5 mg/L were prepared. 0.1 g β-CD/CS porous membranes were added to the PP solution (20 mL) for static adsorption (60 min). Then absorbance of the filtrate was measured, and the Langmuir isotherm adsorption model, the Freundlich isotherm adsorption model and the Temkin isotherm adsorption model were fitted.

### 2.6. Adsorption Kinetics

Adsorption kinetics experiments were carried out by adding using 0.1 g β-CD/CS porous membranes into 20 mL of 2.5, 3.0, 3.5, 4.0 and 4.5 mg/L PP solutions, respectively. Each concentration of PP solution was divided into five groups, the porous membranes were removed after 5, 10, 15, 30 and 60 min of adsorption. Then absorbance of the filtrate was measured, and the Pseudo-first-order kinetic model, the Pseudo-second-order kinetic model, the Double constant kinetic model and the Elovich kinetic model were fitted.

## 3. Results and Discussion

### 3.1. FTIR Results

**Figure 3:**
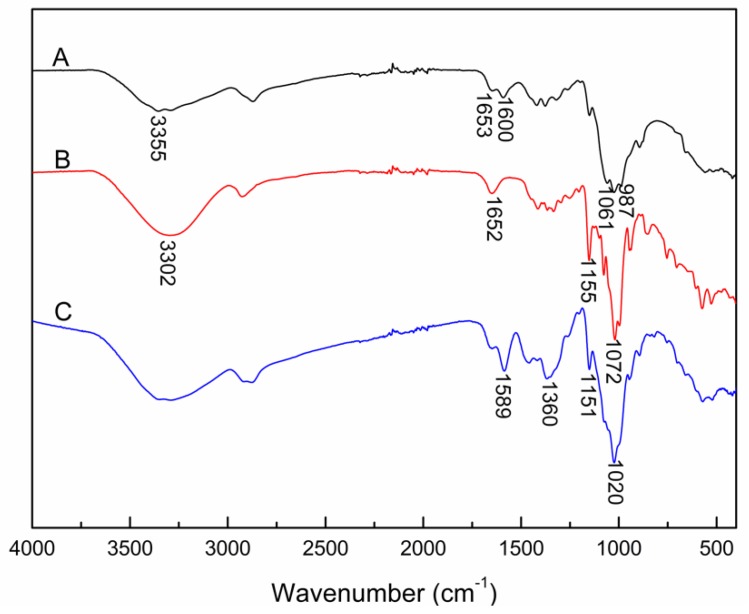
FTIR spectra of different samples. (**A**) CS powder; (**B**) β-CD powder; (**C**) β-CD/CS porous membrane crosslinked for 10 min.

The FTIR spectra of CS powder (A), β-CD powder (B) and β-CD/CS porous membrane (C) crosslinked for 10 min were shown in Figure 3. A broad peak at 3302 cm^−1^ was attributed to the O–H stretching vibrations of β-CD and CS. The peak at 3355 cm^−1^ was the stretching of N–H in CS. The peak strength of β-CD was stronger than that of CS, because β-CD contained more –OH groups. The peak at 1155 cm^−1^ corresponded to the bending vibration of O–H (curve B). While the peak intensity at 3302 cm^−1^ and 1155 cm^−1^ decreased after crosslinking (curve C), which may be caused by –OH groups in β-CD participated in crosslinking reaction. The peak in the range of 1061–987 cm^−1^ was related to the crystallization of CS (curve A) [28]. After crosslinking, the multi-peak became a single peak (curve C) because crosslinking changed the crystallinity of CS. After crosslinking, the intensity of deformation vibration peak of N–H at 1653 cm^−1^ in CS decreased, and a distinct peak was observed at 1589 cm^−1^ which is the characteristic peak of C=N (Schiff base) formed during the crosslinking process. The peak at 1360 cm^−1^ was a signature for stretching vibration of C–O–C (curve C), which verified that the β-CD were crosslinked with glutaraldehyde. Based on the above analysis, the successful crosslinking of CS and β-CD.

### 3.2. Structure Characterization of β-CD/CS Porous Membranes

**Figure 4:**
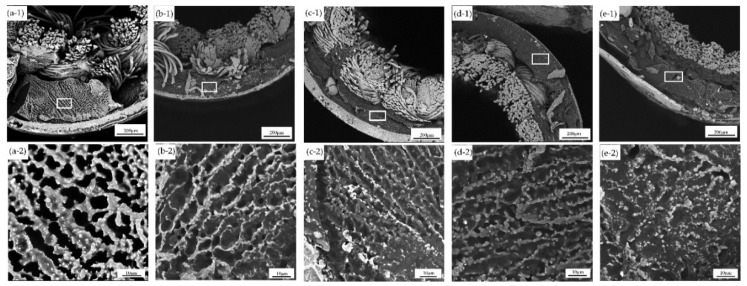
Cross sectional SEM images of knitted tube composite β-CD/CS porous membranes prepared at crosslinking time: 0 s (**a-1**), 10 s (**b-1**), 6 min (**c-1**), 10 min (**d-1**) and 20 min (**e-1**). 0 s (**a-2**), 10 s (**b-2**), 6 min (**c-2**), 10 min (**d-2**) and 20 min (**e-2**) were enlarged drawing of white rectangles, respectively.

In order to maintain the structure of the membrane pores and prevent the pores from collapsing, the membranes were soaked with glycerol and dried before SEM. In addition, the membranes were covered with an epoxy layer for preventing damage of the β-CD/CS porous membranes caused by brittle fracture. The result was displayed in Figure 4. Observing the membranes cross section, the composite membrane structure consisted of a homogeneous membrane layer and a fibrous layer. The homogeneous membrane layer (as shown in the white boxes) was enlarged to show the obvious porous distribution. This could have been related to the extraction of the solvent phase in the NIPS process and the dissolved PEG, as reported in the literature [29]. As the crosslinking time prolonged, the membranes outer skin became denser, because the amine group of CS was reacted with glutaraldehyde to form Schiff base, which formed a crosslinked network structure. The crosslinking density increased with the crosslinking time increased, and the network pores became smaller. After crosslinking, the surface of the membranes was more uneven and folded, which was beneficial to increase the specific surface area and the adsorption contact area.

### 3.3. Mechanical Properties

The mechanical properties parameter of the knitted tube composite β-CD/CS membranes (M-K), crosslinked 10 min knitted tube composite β-CD/CS membranes (M-CK), bare β-CD/CS membranes (M-B) and crosslinked 10 min bare β-CD/CS membranes (M-CB) were listed in Table 1. The results indicated that bare β-CD/CS membrane had poor mechanical performance with tensile strength at 0.13 MPa and elongation at break at 17.36%, while knitted tube composite β-CD/CS membranes displayed a tensile strength of 30.77 MPa and elongation at break of 30.30%. As expected, the coating of β-CD/CS on the knitted tube apparently improved their tensile strength and elongation at break. It revealed that knitted tube as the support materials could bear the more stress loaded on the β-CD/CS membrane and finally possessed the higher mechanical properties. It also can be seen that the tensile strength increased after the crosslinking. After glutaraldehyde crosslinking, the network structure increased and the molecular chains were more closely linked. Thus, the crosslinked membranes showed better flexibility and satisfactory mechanical strength.

### 3.4. Influence of Crosslinking Time on the Hydrophilicity

**Figure 5:**
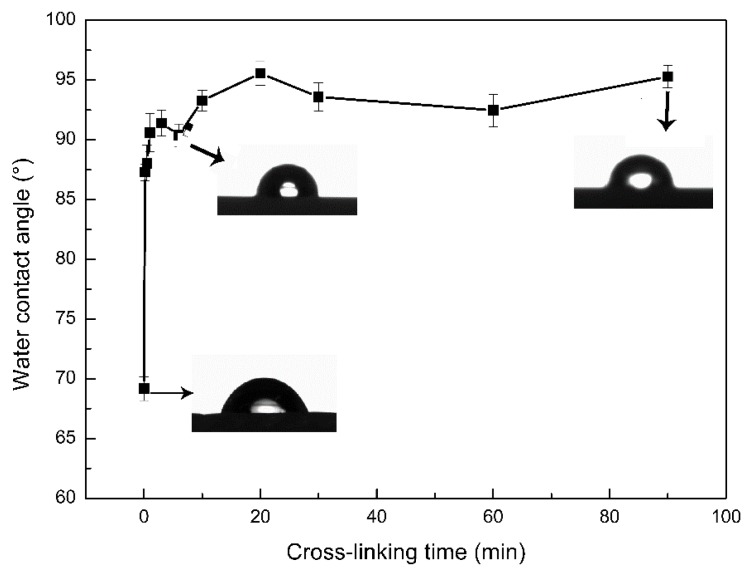
Effect of crosslinking time on contact angle (the three little pictures pointed by the arrows were contact angles figures of crosslinking for 0 min, 6 min, and 90 min, respectively).

The hydrophilicity of the membrane surface was characterized by contact angle. The smaller the contact angle, the better the hydrophilicity of the membrane surface. As the crosslinking time increased from 0 s to 90 min, the contact angle significantly increased from 69° to 95° (Figure 5). The reason was the amine group of CS and the hydroxyl group of β-CD participated in the crosslinking reaction, which made the number of hydrophilic groups reduced. Furthermore, the increase in crosslinking time assisted the porous membranes with a rougher surface morphology (Figure 4), which also contributed to the increase of the contact angle.

### 3.5. Effect of Crosslinking Time on Separation Performance

**Figure 6:**
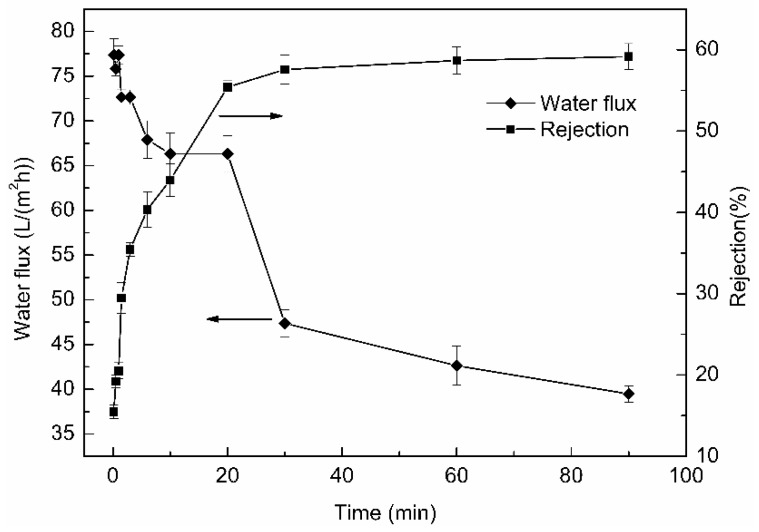
Effect of crosslinking time on water flux and rejection (the arrows pointed to the ordinate of the curve).

The results of rejection performance and water flux were shown in Figure 6. With the crosslinking time increased, water flux of the membranes decreased, while the rejection rate increased. On the one hand, the membranes became denser after crosslinking (Figure 4), resulting in the resistance increased when water and BSA pass through the membranes. On the other hand, the increased hydrophobicity of the β-CD/CS porous membranes caused a decrease of water flux (Figure 5). For these reasons, the water flux became smaller and the rejection rate increased. When the crosslinking time is less than 10 min, the BSA rejection is very low, so the crosslinking time should not be less than 10 min.

### 3.6. Analysis of Adsorption Performance of PP by β-CD/CS Porous Membranes

#### 3.6.1. PP Standard Curve

**Figure 7:**
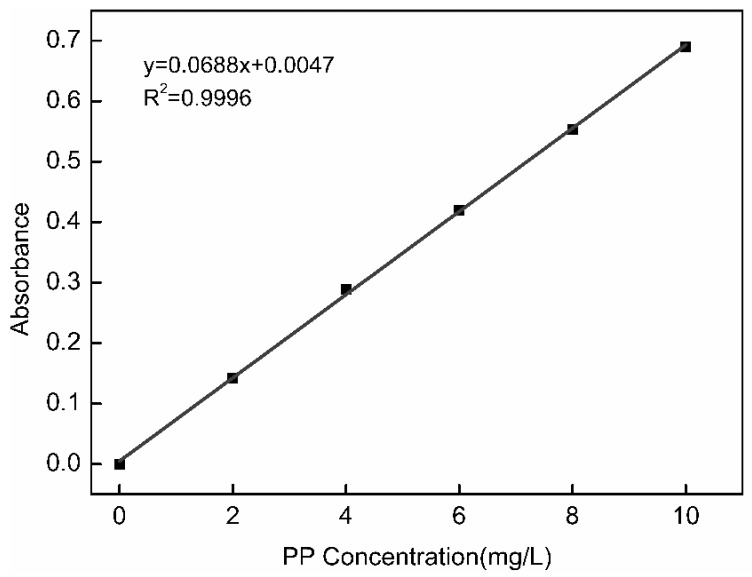
PP solution standard curve.

Figure 7 was the standard curve of PP solution and the Equation (5) was the standard curve formula.

(5) y=0.0688x+0.0047

#### 3.6.2. Effect of Crosslinking Time on Adsorption Rate 

**Figure 8:**
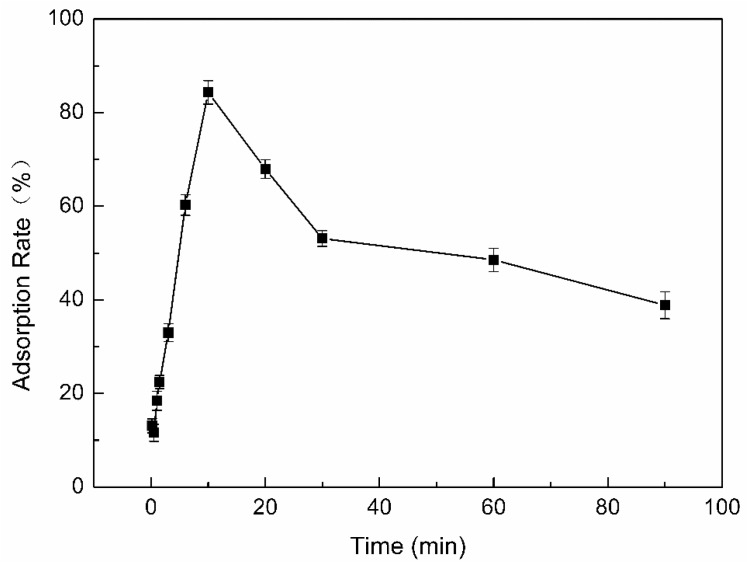
Effect of crosslinking time on adsorption rate.

The adsorption of PP by β-CD/CS porous membranes was mainly attributed to the formation of host-guest inclusion complex between β-CD and PP molecules [30,31]. As demonstrated in Figure 8, when the crosslinking time from 10 s to 10 min, the adsorption rate of PP by β-CD/CS porous membranes has been increasing. The adsorption rate reached the highest at the 10 min, then gradually decreased. A small amount of β-CD was immobilized on surface of the porous membranes under short crosslinking time, which resulted in a low adsorption rate. As the crosslinking time increased, the β-CD immobilized on the surface of the porous membranes gradually increased, and the adsorption rate increased. However, excessive crosslinking increased the brittleness of the membranes and make the structure loose of the porous membrane, this may be caused the surface of the porous membranes detached and led to a decrease in the adsorption rate. In addition, as the crosslinking time increased, a large amount of β-CD participated in the crosslinking reaction, which effected the interaction between PP and β-CD, resulting in the adsorption rate decreased. The optimal crosslinking time of the β-CD/CS porous membranes was 10 min.

#### 3.6.3. Effect of Adsorption Time

**Figure 9:**
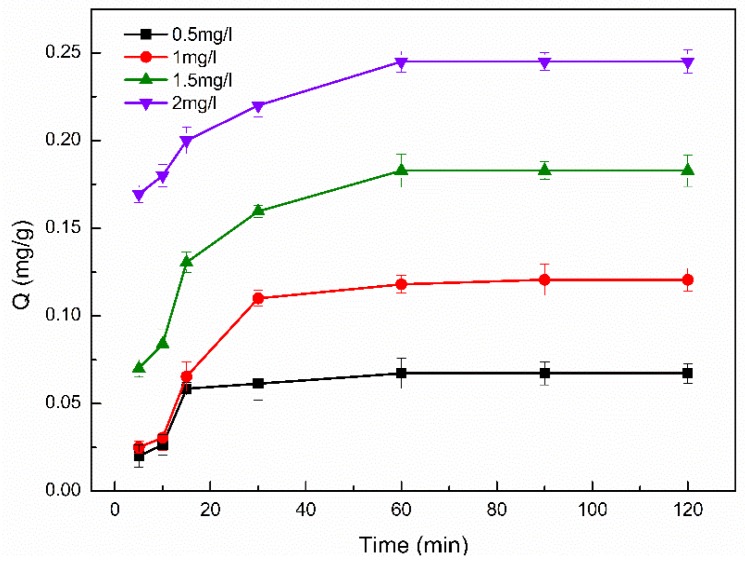
Effect of phenolphthalein (PP) adsorption time (5, 10, 20, 30, 60, 90, 120, 150, 180 min) on adsorption capacity.

The adsorption process took less time to reach equilibrium at low concentrations than that at high concentrations (Figure 9). The adsorption rate of the initial stage was fast, and then became slow. The adsorption sites were not all occupied at the initial stage of adsorption, the PP molecules quickly combined with the adsorption sites, so the adsorption rate was fast. With adsorption time increased the unabsorbed PP molecules in the solution compete with the adsorbed fiercely, resulting in a slow increase in the adsorption capacity. Finally, the adsorption process achieved equilibrium when all the adsorption sites were occupied by the PP molecules. In addition, the adsorption equilibrium time will be extended with the increase of PP solution concentration. The reason was that the amount of adsorption sites for a certain mass of β-CD/CS porous membranes were constant; the intermolecular interaction was weak at low concentrations and the competition for adsorption and desorption was not fierce. Therefore, the PP molecules in the solution quickly combined with the adsorption sites to achieve adsorption equilibrium in a relatively short time. From Figure 9, the different concentrations of PP solutions basically arrived at adsorption equilibrium after 60min. Therefore, 60 min was selected as the optimal adsorption time.

#### 3.6.4. Effect of Initial Concentration

**Figure 10:**
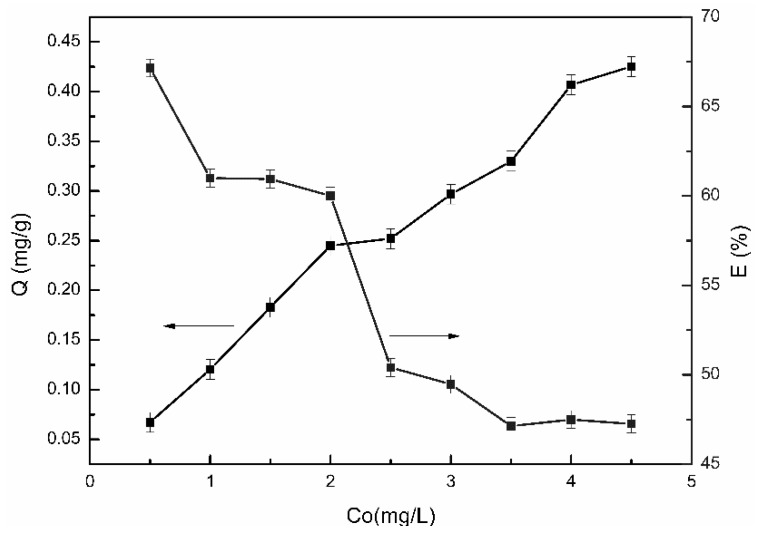
Effect of initial concentration of PP (0.5, 1.0, 1.5, 2.0, 2.5, 3.0, 3.5, 4.0, 4.5 mg/L) on adsorption performance (the arrows pointed to the ordinate of the curve).

Figure 10 displayed the adsorption capacity of the β-CD/CS porous membranes at the different initial concentration of PP. It can be clearly seen that as the initial concentration of the PP solution increased, the adsorption capacity gradually increased but the adsorption efficiency gradually decreased. The reason may be that the number of adsorption sites are constant for a certain β-CD/CS porous membrane. The adsorption sites on the porous membranes were enough at low concentration and the PP molecules can fully combined with β-CD, so the adsorption capacity increased with the concentration increased. However, the PP molecules competed fiercely, and the adsorption sites gradually reached saturation with increasing initial concentration, so the adsorption efficiency decreased.

### 3.7. Adsorption Isotherm

**Figure 11:**
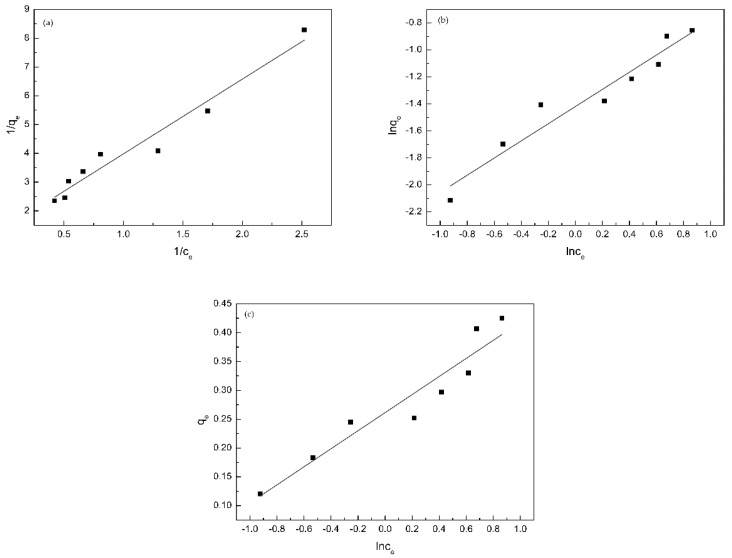
Fits of models for adsorption of PP onto β-CD/CS porous membranes: (**a**) the Langmuir isotherm; (**b**) the Freundlich isotherm and (**c**) the Temkin isotherm.

Adsorption isotherms are used to describe the adsorption state of substances. The Langmuir model describes monolayer adsorption, it is considered that the adsorbent surface is uniform and can adsorb only one adsorbate molecule per adsorption site, which is an ideal state of adsorption [32]. The Freundlich model describes heterogeneous surface adsorption, multi-molecular layer adsorption, interaction between adsorbent and adsorbate and other complex processes [33]. The Temkin model is also suitable for non-uniform surface adsorption and is generally used to describe chemisorption. The experimental data were fitted to the Langmuir isotherm adsorption model (Equation (6)), the Freundlich isotherm adsorption model (Equation (7)) and the Temkin isotherm adsorption model (Equation (8)). The results were shown in Figure 11 and Table 2.

(6) $$\frac{1}{q_{e}}=\frac{1}{b_{L}q_{\max}C_{e}}+\frac{1}{q_{\max}}$$

(7) $$\ln q_{e}=\ln K+\frac{1}{n}\ln C_{e}$$

(8) $$q_{e}=\frac{RT}{b_{T}}\ln A+\frac{RT}{b_{T}}\ln C_{e}$$

where *q_e_* (mg/g) is the equilibrium adsorption capacity, *C_e_* (mg/L) is the equilibrium concentration, *q_max_* (mg/g) is the theoretical saturated adsorption capacity, *b_L_* is the Langmuir constant, *b_T_*, *A* are the Temkin constant, *R* is the gas constant, 8.314J·mol^−1^·*K*^−1^, *T* is the absolute temperature, *K*, *n* are the Freundlich equilibrium constant. It is generally believed that 0.1 < 1/*n* ≤ 0.5, means strong adsorption capacity; 0.5 < 1/*n* ≤ 1, indicates insufficient adsorption capacity; 1/*n* > 1, represents weak adsorption capacity [34].

According to Figure 11 and Table 2, the correlation coefficient (*R*^2^) of the Langmuir model (0.9510) was much higher than that of the Freundlich model (0.9305) and Temkin model (0.9015), which indicated that the monolayer adsorption for PP dominated on the surface of membrane. Therefore, the adsorption of PP was mainly attributed to the formation of host-guest inclusion complex between β-CD and PP molecules, and one PP molecule occupied one β-CD molecule. However, the Freundlich adsorption model is also highly fitted, the result may be induced by the non-uniform distribution and interaction of β-CD on the membranes, which demonstrated that the adsorption process was dominated by monolayer adsorption, and multi-molecular layer adsorption coexisted [35,36,37]. In addition, the value of *1/n* calculated by the Freundlich model was close to 0.5, which manifested that the β-CD/CS porous membranes had strong adsorption capacity for PP.

### 3.8. Adsorption Kinetics

**Figure 12:**
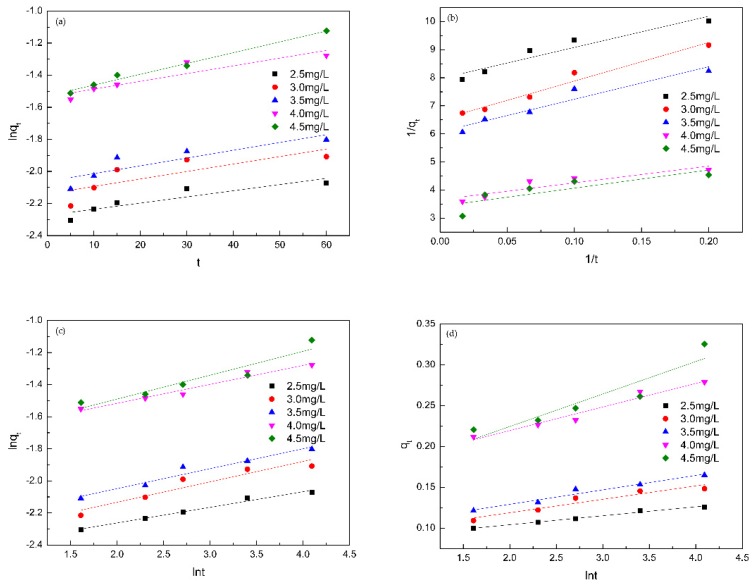
Fits of models for adsorption of PP onto β-CD/CS porous membranes: (**a**) the pseudo first-order kinetic equation; (**b**) the pseudo second-order kinetic equation; (**c**) the Double constant kinetic equation and (**d**) the Elovich equation.

Adsorption kinetics are used to characterize the rate of adsorption process. The Pseudo-first-order kinetic equation mainly describes a simple single reaction and considers that the adsorption process is mainly controlled by diffusion steps. The Pseudo-second-order kinetic equation considers the reaction rate to be determined by the initial concentration, and the degree of chemical reaction between the adsorbate and the adsorbent. The Double constant equation is a dynamic empirical formula suitable for characterizing the complex reaction process. The Elovich equation is also an empirical formula to explain the adsorption process of adsorbents on irregular surfaces with a large number of active adsorption sites, and to illuminate non-ideal adsorption state [38,39,40,41]. The experimental data were fitted to the Pseudo-first-order kinetic model (Equation (9)), the Pseudo-second-order kinetic model (Equation (10)), the Double constant kinetic model (Equation (11)), and the Elovich kinetic model (Equation (12)). The results were exhibited in Figure 12 and Table 3.

(9) $$\ln q_{t}=at+b$$

(10) $$\frac{1}{q_{t}}=\frac{a}{t}+b$$

(11) $$\ln q_{t}=a\ln t+b$$

(12) $$q_{t}=a\ln t+b$$

where *t* (min) is the adsorption time, *q_t_* (mg/g) is the adsorption amount at time *t*, *a*, *b* is the fitting constant.

From Figure 12 and Table 3, we can draw a conclusion that the Double constant kinetic equation and the Elovich kinetic equation had a higher fitting degree, and their fitting coefficients were relatively close, which is also consistent with the existing reports [42]. This result proved that the irregular porous membranes structure (Figure 4) contained a large number of adsorption sites, it also suggested that the adsorption process of PP by the porous membranes was not a single surface adsorption, but a complex process involved host-guest interaction from β-cyclodextrin, non-uniform diffusion and porous network capture.

The parameter *a* in the Double constant equation represents the adsorption rate constant. *a* > 1 indicates that the adsorption rate becomes faster as the adsorption time prolongs. 0 < *a* < 1 indicates that the adsorption rate becomes slower with the extension of the adsorption time, the smaller the *a* the slower the reaction rate [43]. It can be seen from Table 3 that 0 < *a* < 1 and the value of *a* increased as the concentration increased, declaring that the higher the PP concentration, the faster the adsorption rate, but the adsorption rate will slow down over time. This manifested that the whole adsorption process for PP can be divided into two stages: fast adsorption and slow adsorption. β-CD is non-uniformly distributed on the surface and inside of the membranes, the surface of the membranes was uneven and wrinkles, so that the adsorption is not a single surface adsorption, but a result of a combination of multiple adsorptions.

The parameter *b* in the Elovich equation implies the initial rate constant and the larger the *b* the higher the instantaneous adsorption rate [44]. As the initial PP concentration increased, the value of *b* increased (Table 3), which indicated that the adsorption rate became faster as the PP concentration increased. This is consistent with the fitting result of the Double constant equation.

**Figure 13:**
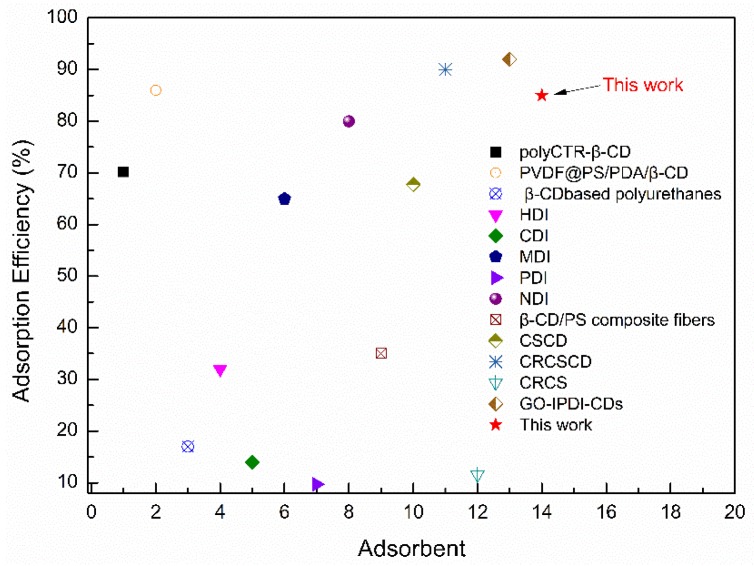
Adsorption capacities of different adsorbents for organic pollutant.

Figure 13 graphically compares the results from this work with those previously reported [20,25,45,46,47,48,49]. The β-CD/CS porous membrane exhibited excellent adsorption efficiency (85%). It can be concluded that β-CD/CS porous membrane is more effective than most typical adsorbents. The reason was the synergistic effect of host-guest interaction from β-cyclodextrin, non-uniform diffusion and porous network capture.

## 4. Conclusions

In this study, knitted tube composite β-CD/CS porous membranes with separation and adsorption functions were prepared by NIPS method. The FTIR demonstrated that the β-CD and CS had been successfully crosslinked. The membrane became denser, uneven, and folded, which provided a large contact area for adsorption. With the increase of crosslinking time, the water flux decreased, whereas the contact angle and rejection rate increased. Knitted tube as reinforcement made the membrane have better mechanical properties. The strength and the elongation at break were 236 and 1.7 times higher than these of bare β-CD/CS porous membranes, respectively and the membranes showed a higher tensile strength after crosslinking. As the crosslinking time increased, the adsorption rate first increased and then decreased, and the adsorption efficiency reached a maximum of 85% which was higher than that of many other adsorbents with β-CD. Adsorption isotherm showed that the adsorption process of PP by porous membranes were dominated that the coexistence of monolayer adsorption and multi-layer adsorption. Adsorption kinetics revealed that the adsorption process followed the Double constant model and the Elovich model, indicating that the adsorption process was more complicated. The adsorption mechanism involved the formation of inclusion complexes between β-CD and PP, accompanied with the non-uniform diffusion and porous network capture.

## Figures and Tables

**Table 1:** Mechanical properties parameters of membranes.

Membranes	Tensile Strength/MPa	Elongation at Break/%
M-K	30.77	30.30
M-CK	33.97	32.42
M-B	0.13	17.36
M-CB	0.19	21.53

**Table 2:** Isothermal model parameters of β-CD/CS porous membrane.

Parameters	Langmuir	Freundlich	Temkin
Constants	*q_m_* = 0.7242	*n* = 1.5716	*b_T_* = 15821
	*b_L_* = 0.5313	*k* = 0.2417	*A* = 5.3069
*R* ^2^	0.9510	0.9305	0.9015

**Table 3:** Kinetic equation fitting results of β-CD/CS porous membranes.

Equation	Parameters	*C_o_* (mg/L)				
		2.5	3	3.5	4	4.5
Pseudo-first-order Equation	*a*	0.0039	0.0047	0.0049	0.0048	0.0067
	*b*	−2.2744	−2.1400	−2.0622	−1.5346	−1.5286
	*R* ^2^	0.7594	0.5231	0.6970	0.8101	0.9807
Pseudo-second-order Equation	*a*	11.1047	13.7550	11.5965	5.9720	6.4760
	*b*	7.9696	6.5067	6.0742	3.6564	3.4184
	*R* ^2^	0.8936	0.9613	0.9081	0.7814	0.6053
Double Constant Equation	*a*	0.0973	0.1180	0.1272	0.1244	0.1489
	*b*	−2.4566	−1.7519	−2.8375	−2.2967	−1.7872
	*R* ^2^	0.9992	0.9526	0.8716	0.9374	0.8748
Elovich Equation	*a*	0.0110	0.0164	0.0177	0.0398	0.0289
	*b*	0.0822	0.0860	0.0938	0.1450	0.1618
	*R* ^2^	0.9821	0.8905	0.9496	0.8310	0.9469
